# CpG-induced immune responses *via* DNA micelles, gold nanoparticles, and liposomes

**DOI:** 10.1039/d5nh00726g

**Published:** 2025-11-27

**Authors:** Hongyan Li, Hae-Bin Park, Haejoo Kim, Sang Hak Lee, Andreas Herrmann, Jun-O Jin, Minseok Kwak

**Affiliations:** a DWI – Leibniz Institute for Interactive Materials Forckenbeckstraße 50 52074 Aachen Germany; b Institute of Technical and Macromolecular Chemistry, RWTH Aachen University Worringerweg 2 52074 Aachen Germany; c Department of Microbiology, Brain Korea 21 project, University of Ulsan College of Medicine, ASAN Medical Center Seoul 05505 South Korea; d Department of Chemistry and Industry 4.0 Convergence Bionics Engineering, Pukyong National University Busan 48513 Republic of Korea mkwak@pukyong.ac.kr; e Department of Chemistry and Institute for Future Earth, Pusan National University Busan 46241 Republic of Korea

## Abstract

CpG oligodeoxynucleotides (CpG ODNs) are well-known adjuvants that induce innate immunity, particularly dendritic cell activation, by stimulating Toll-like receptor 9. However, the stimulatory efficacy of CpG ODNs is limited by their negative charge, which causes electrostatic repulsion from the cell membrane and hinders cellular uptake. In addition, CpG ODNs are rapidly degraded by nucleases under physiological conditions. To address these challenges, various nanoparticle (NP)-based delivery systems have been developed and applied across biomedical fields. Although various types of NPs have been utilized, the relationship between their physical properties and CpG delivery efficiency remains under investigation. In this study, we selected three commonly used and well-established nanocarriers—DNA micelles, gold nanoparticles (AuNPs), and liposomes—which differ in size and rigidity and are known for their effectiveness in drug delivery. We aim to evaluate and compare the *in vivo* delivery efficiency and immunostimulatory activity of these NPs when functionalized with CpG ODNs, thereby providing insights into how NP properties influence CpG-mediated immune activation.

## Abstract 2

This study compares the immunostimulatory efficiency among three different CpG-loaded nanocarriers: DNA micelles, liposomes, and gold nanoparticles.

## Graphical abstract

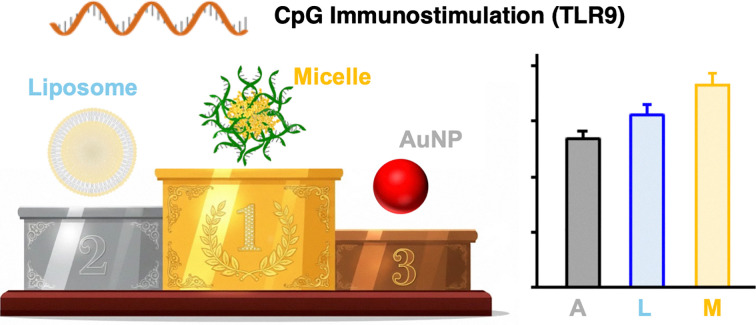

**New concepts**

This study compares three nanocarrier platforms—DNA micelles, gold nanoparticles, and liposomes—for the *in vivo* delivery of immunostimulatory CpG oligodeoxynucleotides. Previous research has primarily evaluated the effect of a specific property of nanoparticles (NPs), such as size or surface charge, on drug delivery and immune activation. In contrast, our study directly compares three distinct NP platforms. It demonstrates that their overall physicochemical properties—size, surface charge, hydrophobicity, and CpG surface density—collectively influence Toll-like receptor 9 (TLR9)-mediated immune responses. Notably, despite having a highly negative surface charge, DNA micelles induced the strongest immune responses. This effect is attributed to their enhanced cellular uptake, resulting from hydrophobic interactions between the lipid-modified DNA and the cell membrane, as well as a suitable surface density of CpG that promotes more effective recognition with TLR9. This study demonstrates that nanoparticle properties significantly impact immune activation *in vivo*, providing valuable insights for enhancing nanoparticle-based CpG delivery systems. Emphasizing the role of multiple interrelated design parameters in achieving effective immune responses, the findings provide new insights applicable to nanomedicine, immunotherapy, and vaccine development.

## Introduction

Immunotherapy exploits natural defensive mechanisms available in the body to fight cancer, for example, by targeting cells and biomolecules to facilitate and/or redirect the immune response toward the malignant process.^1^ Unmethylated cytosine–phosphate–guanine (CpG) motifs, frequently present in bacterial and viral DNA, have long been recognized as activating mammalian immune cells, such as dendritic cells (DCs), macrophages, and B cells.^2,3^ This activation depends on its interactions with Toll-like receptor 9 (TLR9), which is followed by the production of proinflammatory cytokines, including interleukin (IL)-6, IL-12, interferon (IFN)-α, and TNF-α, as well as the upregulation of major histocompatibility complex (MHC) and co-stimulatory molecules, such as CD80, CD86, and CD40.^4,5^ As CpG activates antigen-presenting cells (APCs), synthetic CpG oligodeoxynucleotide (CpG ODN) holds tremendous potential as an immunotherapeutic reagent for the treatment of many diseases, including cancers, allergies, and infectious diseases.^6^ Nevertheless, CpG ODN has to enter intracellular vesicles to function well because TLR9 is exclusively expressed on the endoplasmic reticulum, endosomes, lysosomes, *etc.*^7^ However, the application of CpG ODNs is challenged by several limitations. The negative charge of CpG ODNs leads to electrostatic repulsion with similarly charged cell membranes, thereby reducing cellular uptake efficiency.^8^ Moreover, naked CpG ODNs are susceptible to enzymatic degradation by DNase.^9–11^ To address these challenges, nanoparticle (NP)-based delivery systems have been developed. Depending on the strategy, CpG ODNs can be either encapsulated within NPs or displayed on their surfaces to enhance immunostimulatory effects.^12,13^ These NPs improve the biostability of CpG ODNs by protecting them from nuclease-mediated degradation and enhancing cellular uptake.

**Fig. 1:**
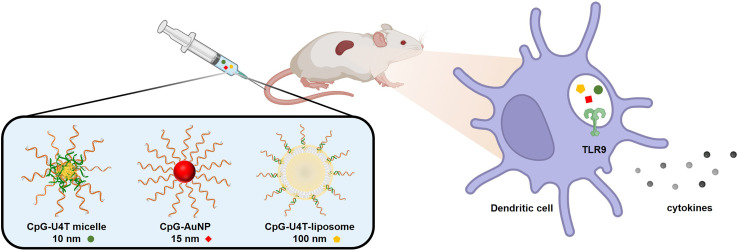
Schematic illustration of immunomodulatory nanoparticle delivery *in vivo*. Different types of CpG-functionalized nanoparticles are administered to mice *via* intravenous injection. The nanoparticles are taken up by dendritic cells in the spleen, where CpG oligonucleotides engage TLR9 in endosomes. This interaction triggers downstream signalling cascades, leading to dendritic cell activation and cytokine secretion.

In this study, we employed three types of NPs—DNA micelles, gold NPs (AuNPs), and liposomes—as delivery platforms of CpG ODNs (Fig. 1). DNA micelles are nucleic acid-based micelle nanostructures composed of synthetic amphiphilic DNA strands. CpG ODNs can be easily decorated on the micelle surface *via* sequence-specific base-pairing. Our previous studies have shown that DNA micelles exhibit low cytotoxicity due to their nucleic acid composition and efficient drug delivery capabilities, even in the absence of transfection reagents.^14^ Furthermore, the micellar structure and the modification of the phosphate backbone enhanced resistance against DNase-mediated degradation.^15,16^ Due to these properties, DNA micelles have been utilized for the delivery of various functional molecules, including immune adjuvants,^16^ gene-silencing agents,^14^ peptides,^15^ and fluorescent probes.^14^ AuNPs were selected as another carrier due to their excellent biocompatibility, high cellular uptake efficiency, and well-established surface modification chemistry.^17,18^ Thiolated CpG can be functionalized onto AuNPs *via* strong Au–S bonds.^19,20^ Moreover, a dense shell of CpG ODNs can form on the surface of AuNPs, which enhances nuclease resistance by limiting nuclease accessibility.^21^ A third platform is the liposome, a self-assembled vesicle composed of phospholipids with a bilayer membrane structure. Liposomes have been widely used in pharmaceutical and cosmetic applications due to their ability to encapsulate a wide range of materials. They protect encapsulated molecules from enzymatic degradation, prolong circulation time, and enable controlled release, while exhibiting high biocompatibility and safety.^22,23^ In addition to encapsulating CpG ODNs in their inner aqueous core, the effective anchoring of CpG molecules onto the outer lipid bilayer surface has also been recently developed and applied.^24^ Therefore, amphiphilic DNA can be anchored to the liposome lipid bilayer, exposing CpG on the surface *via* hybridization with eCpG. In this way, CpG can be functionally displayed on the surfaces of DNA micelles, AuNPs, and liposomes using appropriate strategies, enabling their application as CpG delivery platforms.

NPs have been widely utilized for drug delivery due to their diverse physicochemical and structural properties, allowing for the selection of specific applications. However, the therapeutic efficacy of NP-based delivery systems depends on their ability to reach target sites through blood circulation. In the case of CpG ODNs, efficient delivery into APCs is crucial, as recognition by TLR9 occurs within the endosomal structure of the cells.^25,26^ Various factors, such as particle size, shape, surface charge, and hydrophobicity, can significantly influence cellular uptake.^27,28^ Among these, particle size is considered an important parameter that affects immunological responses,^29^ and several studies have suggested that the pathway through which NPs reach APCs differs according to the particle size.^30^ However, according to related studies, the correlation between NP size and immunostimulatory efficacy has been inconsistent. Therefore, we investigated the *in vivo* cellular delivery and immune activation efficiency of CpG-functionalized NPs with distinct physical characteristics and sizes—namely, DNA micelles (∼10 nm), gold nanoparticles (∼15 nm), and liposomes (∼100 nm) (Fig. 1).

## Results and discussion

### CpG-functionalized NP characterization

**Fig. 2:**
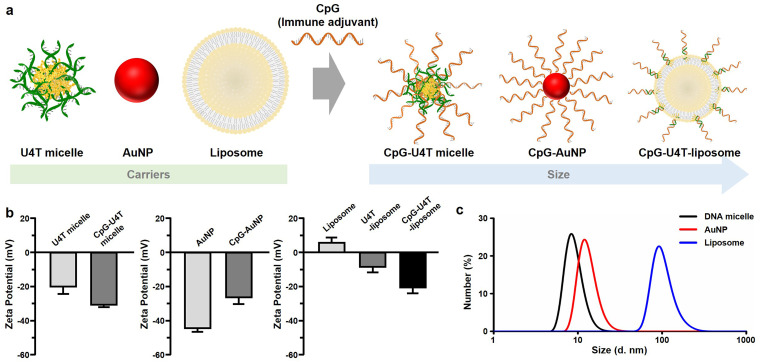
Design and characterization of carriers for CpG delivery. (a) Schematic illustration of three types of nanoparticle platforms functionalized with CpG oligonucleotides. (b) Zeta potential analysis of bare and CpG-functionalized nanoparticles. (c) Hydrodynamic diameters of nanoparticles. Black: DNA micelle, Red: AuNP, Blue: liposome.

We prepared three types of NPs – DNA micelles, AuNPs, liposomes – for evaluation of the immune stimulatory effect of CpG in splenic DCs *in vivo* (Fig. 2a). CpG-incorporated DNA micelles, formed by lipid-modified DNA (U4T), has been reported to induce potent immune activation in both naïve and tumor-bearing mice.^31^ U4T is an amphiphilic DNA composed of four consecutive lipid-modified uracils and fourteen unmodified nucleotides, which enables its self-assembly into micellar structures in aqueous solution^32^ (Fig. S1a), as confirmed by transmission electron microscopy (TEM) (Fig. S1b). To decorate the micelle surface with CpG, we extended its 3′-end with a sequence complementary to U4T (eCpG), enabling hybridization between CpG and the micelle (Fig. S1a). Additionally, the phosphodiester backbone of CpG was replaced with a phosphorothioate linkage to improve nuclease resistance.^15^ CpG-U4T micelles were assembled at a 1 : 2 molar ratio (CpG : U4T), which showed maximal immunostimulatory activity *in vitro* and *in vivo*, with the U4T concentration maintained above the critical micelle concentration.^14,33^*Via* the base-pairing property of DNA, when eCpG (40 µM) and DNA micelles (80 µM) were mixed at a 1 : 2 molar ratio, all eCpG strands were incorporated into the DNA micelle.^15^ Therefore, the amount of CpG included in the CpG-U4T micelle was equivalent to the amount of CpG added during the preparation of the micelles (40 µM).

As a second delivery platform, we prepared CpG-capped AuNPs (CpG-AuNPs), based on the well-established Au–S chemistry^34^ (Fig. S1c). AuNPs are widely used in drug delivery due to their versatility of surface functionalization and colloidal stability.^35^ In this study, we prepared 15 nm AuNPs, characterized by UV-vis spectroscopy and TEM (Fig. S1d and e). Moreover, the CpG oligonucleotide used in this study was a thiolated CpG, which was modified at the 5′-end with a thiol group, and a 20-thymine (T) spacer was inserted to optimize the presence of CpG on the AuNP surface. To quantify the amount of thiolated CpG on the surface of AuNPs, the CpG strands were released from the AuNPs by adding dithiothreitol, followed by absorbance measurement. The quantification result revealed that approximately 100 thiolated CpG strands were attached to each AuNP. Accordingly, the CpG concentration was fixed at 40 µM for *in vivo* experiments.

Finally, we constructed CpG-incorporated liposomes (Fig. S1f). Liposomes are spherical vesicles composed of a phospholipid bilayer and are widely used as a drug delivery carrier. Due to U4T containing hydrophobic lipid moieties, it can be co-assembled with the liposomal components to form U4T-incorporated liposomes (U4T-liposomes). CpG was then hybridized to the U4T on the liposome surface *via* eCpG, enabling surface presentation similar to that of micelles (Fig. S1f). To prepare CpG-U4T-liposome, we first formed U4T-incorporated liposomes (U4T-liposome) by mixing DOPC : DOPE : cholesterol : U4T at a molar ratio of 3 : 2 : 2 : 1, followed by hybridization with eCpG. The preparation of each CpG-incorporated NP was confirmed by zeta potential measurements (Fig. 2b and Table S1). Due to the negatively charged phosphate backbone of the CpG oligonucleotide, DNA micelles and liposomes exhibited more negative zeta potentials compared to their bare NPs. In contrast, AuNPs exhibited a reduced negative zeta potential after CpG functionalization, which is attributed to the replacement of citrate—also negatively charged—on the surface of bare AuNPs. Nevertheless, all types of NPs showed distinct shifts in zeta potential upon CpG decoration, indicating that CpG was successfully present on their surfaces. To quantify the amount of CpG loaded into liposomes, CpG-U4T-liposomes were prepared using ATTO590-labeled eCpG (ATTO590-eCpG). A standard calibration curve was generated by measuring the fluorescence intensity of ATTO590-eCpG strands. The fluorescence intensity of the prepared ATTO590-CpG-U4T-liposomes was then measured to calculate the amount of CpG incorporated into the liposomes (Fig. S1g). Based on the quantification analysis, the CpG concentration was adjusted to 40 µM for *in vivo* experiments.

The three types of NPs differ in size and rigidity. As shown in Fig. 2c and Table S2, their sizes increase in the order of DNA micelles (∼10 nm), gold nanoparticles (∼15 nm), and liposomes (∼100 nm). In terms of rigidity, DNA micelles and liposomes are softer compared to AuNPs. From these distinct physical properties, this study aims to evaluate how each NP platform performs in delivering CpG effectively.

### Activation of splenic DCs in the mouse *in vivo*

Previous studies have demonstrated that CpG-incorporated NPs, including DNA micelles,^33^ AuNPs,^36,37^ and liposomes,^38^ can be efficiently delivered to both BMDCs and splenic DCs. The immunostimulatory effect of CpG has been confirmed by inducing various cytokines, including TNF-α, IL-6, IFN-α, and IL-12p70. Additionally, CpG-incorporated NPs have been shown to accumulate at high concentrations in the spleen and liver *in vivo*.^14,39,40^ Furthermore, in a previous study using CpG-U4T, dose-dependent experiments identified an optimal CpG concentration of 40 µM within the U4T construct.^33^ Therefore, in the present study, the amount of CpG in all nanoparticle formulations was standardized to 40 µM.

**Fig. 3:**
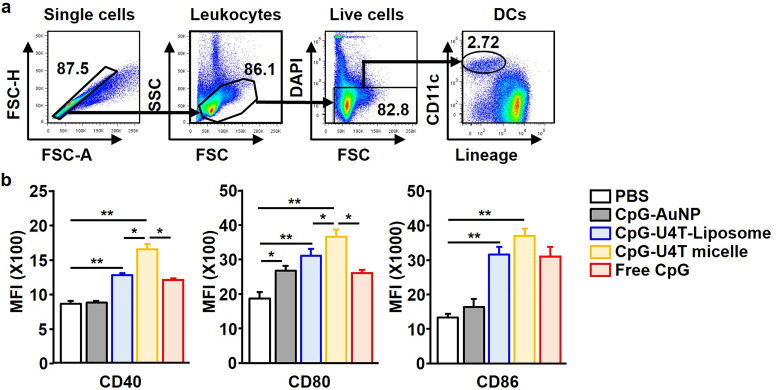
Spleen DC activation *in vivo* by administration of immunostimulatory NPs. Three different CpG NPs (AuNPs, liposomes, and micelles) were injected intravenously (*i.v.*) into C57BL/6 mice with 100 µL eCpG (40.8 µM) doses. Eighteen hours after the injection, the spleen was harvested. (a) The definition of splenic DCs, defined as lineage – CD11c^+^ in the live leukocyte population, was analysed by flow cytometry. (b) Mean fluorescence intensity (MFI) of CD40, CD80, and CD86 on splenic DCs was measured by flow cytometry. Data are representative and the average of six independent samples (*n* = 6, total of two independent experiments). *p < 0.05; **p < 0.01 by Student's *t* test.

To determine whether different formulations of NPs have distinct effects on the immune response, NPs were administered intravenously (*i.v.*) to 6-week-old C57BL/6 mice. After 18 h, lineage–CD11c+ cells in live leukocytes were identified as splenic DCs (Fig. 3a). Administration of CpG-loaded micelles led to an increase in the surface expression levels of CD40, CD80, and CD86 in splenic DCs (Fig. 3b). Liposomes considerably increased the expression of co-stimulators in splenic DCs, although less significantly than micelles. In contrast, AuNPs showed lower DC activation ability than free CpG (Fig. 3b).

**Fig. 4:**
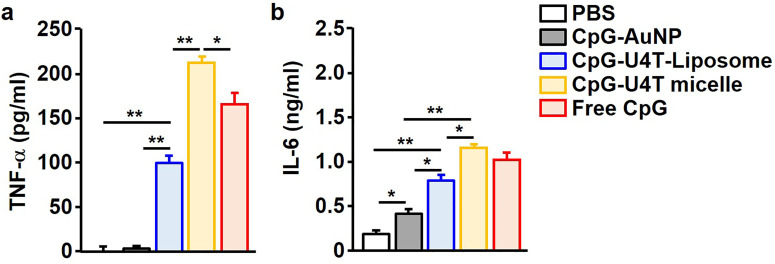
Immunostimulatory NPs promote sera concentration of pro-inflammatory cytokines. C57BL/6 mice were injected with the indicated NPs, and cytokine levels in serum were measured 18 h after injection. (a) TNF-α concentration in sera. (b) IL-6 concentration in sera. Data are representative and the average of six independent samples (*n* = 6, total of two independent experiments). *p < 0.05; **p < 0.01 by Student's *t* test.

Another immune-stimulating ability of CpG is to increase the production of pro-cytokines. Therefore, we further determined their production levels in serum by injecting immunostimulatory NPs. Eighteen hours after injection, immunostimulatory micelles induced a dramatic increase in serum TNF-α and IL-6 production levels compared with other NPs, which showed a similar trend as the expression of surface activation markers. AuNPs also showed weak activity in the production of pro-inflammatory cytokines, similar to the weak induction of activation marker elevation in DCs (Fig. 4).

Among the three types of NP platforms evaluated, DNA micelles exhibited the most CpG-induced immune activation. Several factors caused this result. As shown in Fig. 2b, all CpG-functionalized NPs exhibited negative surface charges, leading to electrostatic repulsion with the cell membrane. This repulsion reduces the cellular uptake of CpG, thereby limiting its interaction with TLR9 within the endosome of DCs.^41^ Such electrostatic hindrance reduces the immune activation induced by CpG-functionalized AuNPs and liposomes. Nevertheless, DNA micelles, which exhibited the most negative zeta potential among the NPs, induced higher immune responses than pristine CpG. This phenomenon is attributed to the enhanced cellular uptake of DNA micelles, which results from hydrophobic interactions between the lipid-modified DNA and the cell membrane.^16^

Distinct surface CpG density might be one possibility why these different NPs exhibited varied immunostimulatory responses. Surface crowding of CpG on AuNPs was the highest (0.19 molecules per nm^2^) as compared with that on the liposome structure (0.01 molecules per nm^2^) and micelle structure (0.07 molecules per nm^2^). Crowded CpG molecules on AuNPs might give rise to insufficient binding of CpG with the TLR9 receptor after entering the endosome. Although a 20-T spacer was employed as a spacer between the CpG segment and the AuNP surface, in the hope of reducing the steric hindrance of CpG to facilitate its interaction with the TLR9 receptor, this reduction might still not be sufficient. A more flexible TEG^36^ or PEG^42^ spacer might still be needed to provide greater rotation and mobility of the extended CpG strands on the AuNPs surface. Although there are *in vitro* reports suggesting that a 15-nucleotide spacer helps with stimulatory activities in TNF-α secretion as compared with free CpG,^37^ we still believe the necessity of a more flexible segment, as *in vivo* is much more complicated than *in vitro*.

Overall, the immunostimulatory efficacy of CpG-functionalized nanoparticles *in vivo* is influenced by a complex interplay of factors, including particle size, surface charge, and CpG surface density. Therefore, the balance of these physical properties plays an important role in determining the effect of CpG-induced immune activation.

## Conclusions

In this study, we evaluated the *in vivo* immunostimulatory effects of CpG-functionalized nanoparticles—DNA micelles, AuNPs, and liposomes. Among the three types of platforms, DNA micelles showed the most immune activation, as evidenced by the enhanced activation of spleen DCs and cytokine production. Despite their highly negative surface charge, DNA micelles exhibited efficient cellular uptake, due to hydrophobic interactions between lipid-modified DNA and the cell membrane. In contrast, CpG-AuNPs and CpG-liposomes showed reduced immune activation compared with pristine CpG, which was attributed to electrostatic repulsion and surface CpG density. These results highlight that complex physicochemical properties, such as particle size, surface charge, CpG density, and membrane interaction, influence the immunostimulatory effect of CpG-functionalized NPs. Our findings offer insights into the design of effective NP-based CpG delivery platforms.

## Materials and methods

### Materials

Unless otherwise noted, all chemicals and reagents purchased from commercial suppliers were used without further purification. 1,2-Dioleoyl-*sn-glycero*-3-phosphocholine (DOPC), 1,2-dioleoyl-*sn-glycero*-3-phosphoethanolamine (DOPE), cholesterol (plant-derived), and polycarbonate membranes with a diameter of 100 nm were purchased from Avanti Polar Lipids, Inc. HAuCl_4_, sodium citrate, dithiothreitol (DTT), and Histopaque®-1077 were purchased from Sigma-Aldrich. Fetal bovine serum and phosphate-buffered saline (PBS) buffer were received from Gibco™. Antibody CD40 (3/23), CD80 (16-10A1) and CD86 (GL-1) were received from eBioscience™. 7-Aminoactinomycin D (7-AAD) and ELISA kits were purchased from BioLegend, Inc. All oligonucleotides without lipid modification were purchased from Biomers.net GmbH and purified by HPLC.

### Mice

C57BL/6 mice were obtained from Orient Bio, Inc. (South Korea). Mice were housed in a pathogen-free environment and used under appropriate institutional guidelines.

### DNA

Lipid-modified DNA was synthesized and characterized in previous reports.^32^ DNA sequences used in this work are listed in Table 1.

**Table 1:** DNA sequences used in the study. **U̲** represents dodecyne-modified deoxyuridine nucleotide. In the sequence, an asterisk (*) indicates a phosphorothioate modification at the corresponding nucleotide

Name	Sequence (5′ to 3′)
U4T	**U̲U̲U̲U̲**GCGGATTCGTCTGC
Thiol-CpG	Thiol C6-T*C*C*A*T*G*A*C*G*T*T*C*C*T*G*A*C*G*T* T*T*T*T*T*T*T*T*T*T*T*T*T*T*T*T*T*T*T
eCpG	T*C*C*A*T*G*A*C*G*T*T*C*C*T*G*A*C*G*T*T* GCAGACGAATCCGC
ATTO590-eCpG	ATTO590-TCCATGACGTTCCTGACGTTGCAGACGAATCCGC
Free CpG	T*C*C*A*T*G*A*C*G*T*T*C*C*T*G*A*C*G*T*T

### CpG-incorporated DNA micelle preparation

A U4T and eCpG mixture was prepared by mixing 80 µM of U4T and 40 µM of eCpG in the presence of 1× PBS (pH 7.5 at 25 °C) and MgCl_2_ (2 mM). The solution was denatured at 95 °C for 5 min and subsequently annealed from 80 °C to 20 °C with −1 °C min^−1^.

### CpG-gold nanoparticle preparation

Prior to the preparation of CpG-AuNPs, citrate-capped AuNPs of about 15 nm were synthesized following a reported protocol.^43^ Briefly, 225 mL of 1 mM HAuCl_4_ (88.61 mg) in Milli-Q water was loaded into the rounded-bottom two-neck flask, then the flask was placed on a hot plate to heat the solution until reflux. Afterwards, 25 mL of 38.8 mM sodium citrate (285 mg) was quickly added and allowed to reflux for 30 min with vigorous agitation. Subsequently, the solution was cooled down to room temperature under stirring. The concentration of AuNP solution was determined by absorption at 520 nm with a corresponding extinction coefficient of 2.33 × 10^8^ M^−1^ cm^−1^.

To conjugate thiol-CpG to the AuNPs surface, a 300 µL AuNP solution was mixed with 40 µL thiolated CpG (200 µM in Milli-Q water) for 10 min at room temperature, then 108.3 µL of 100 mM Tris buffer (pH 3) was quickly added and incubated at room temperature for a further 60 min. After that, the solution was subjected to 30 min centrifugation at 15 000 rpm. Supernatant was removed, and fresh PBS buffer was added and rinsed 3 times to remove any unconjugated thiolated CpG. After purification, the AuNPs pellet was re-dispersed in 1 mL of 1× PBS buffer.

To quantify the amount of thiolated CpG on the AuNP surface, 5 µL of the AuNP solution was diluted with 90 µL of Milli-Q water and then mixed with 5 µL of DTT solution (1 M in Milli-Q water). After incubation at 60 °C for 1 h, the solution was centrifuged at 15 000 rpm for 30 min, and the absorbance of the supernatant at 260 nm was measured to quantify the released thiolated CpG strands from the AuNP surface. Approximately 100 thiolated CpG strands were conjugated to each AuNP. The final concentration of thiolated CpG in the CpG-AuNP solution was adjusted to 40.8 µM with 1× PBS buffer for *in vivo* experiments.

### CpG-U4T-liposome preparation

For quantification of eCpG that could be loaded on the liposome surface, 400 µL of a mixture of DOPC, DOPE, and cholesterol (2 : 1 : 1) in ethanol (DOPC and DOPE total concentration was 10.08 mM) was mixed with 32.9 nmol dry U4T. The molar ratio between liposome lipids (DOPC and DOPE) and U4T was 123. Ethanol was evaporated by a dry N_2_ stream. The dried lipid film was rehydrated with 1× PBS. Lipid emulsion was sonicated for 5 min, then subjected to 5 freeze–thaw cycles and 21 times extrusion through a 100 nm polycarbonate membrane by a Mini Extruder (Avanti Polar Lipids). As a control, a liposome anchored with the U4T was also prepared as a U4T-liposome. After extrusion, 100 µL of U4T-liposome was mixed with 4.08 µL of eCpG (1mM) and hybridized using a thermal gradient (40 °C, 30 min; −1 °C min^−1^ until 4 °C). After hybridization, liposomes were transferred to a Vivaspin column (Viva 6, 300k Mw cut-off) and rinsed 3 times with 1× PBS buffer to remove any non-hybridized eCpG from the solutions. Then, 4 mL of 1× PBS was added to the CpG-U4T-liposome solution.

To quantify the amount of CpG loaded in CpG–U4T–liposomes, 100 µL of U4T-liposome solution was mixed with 4.08 µL of ATTO590-eCpG (1 mM) and hybridized using a thermal gradient (40 °C for 30 min, followed by cooling at −1 °C min^−1^ to 4 °C). After hybridization, the mixture solution was transferred to a Vivaspin column (Viva 6, 300 kDa MW cut-off) and rinsed three times with 1× PBS to remove unhybridized ATTO590-eCpG. Subsequently, 4 mL of 1× PBS was added, and the fluorescence intensity of the ATTO590-CpG-U4T-liposome solution was recorded using a SpectraMax® M3 Multi-Mode Microplate Reader. A calibration curve of ATTO590-eCpG was generated by measuring the fluorescence intensity of ATTO590-eCpG solutions at various concentrations in 1× PBS. The amount of ATTO590–eCpG incorporated into liposomes was determined from this calibration curve and compared with the input amount to calculate the hybridization efficiency. For *in vivo* experiments, eCpG was hybridized with all anchored U4T strands in the liposomes, and the final eCpG concentration was adjusted to 40.8 µM with 1× PBS buffer.

### TEM

5 µL of AuNPs or U4T micelle solution was deposited on a glow-discharged holey carbon-coated grid. The excess of solution was blotted off with a filter paper. For the micelle solution, the grid was further stained with 2% uranyl acetate solution. After drying overnight, samples were examined using a Libra 120 transmission electron microscope (Carl Zeiss, Germany) with 120 kV accelerating voltage.

### DLS

Nanoparticles in 1× PBS buffer were filtered using 0.45 µm syringe filters, and their size was measured using a Zetasizer Ultra (Malvern Panalytical) at 25 °C. The nanoparticle concentration was adjusted so that the CpG concentration was 40 µM, and diameters were averaged from the number distribution by three measurements.

### Zeta potential

500 µL of nanoparticles in 1× PBS buffer was added to the zeta cell and measured by Zetasizer Ultra (Malvern Panalytical) at 25 °C. The nanoparticle concentration was adjusted so that the CpG concentration was 40 µM, and the surface zeta potential was averaged from three measurements.

### Surface coverage calculation

The number of lipids per liposome was estimated as outlined below. The total area of the inner and outer leaflets of a spherical liposome is:
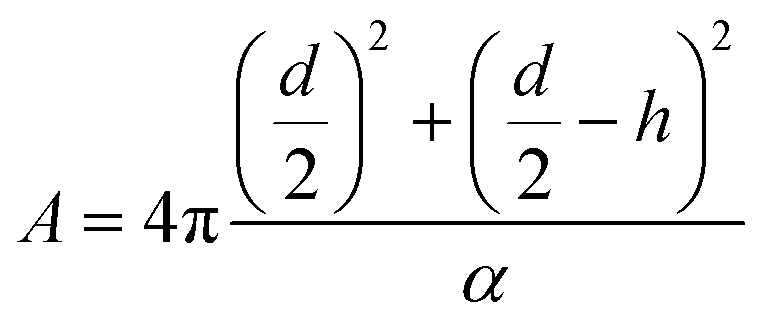


The diameter of the liposome (outer surface) is the thickness of the bilayer, about 5 nm, and the lipid head group area. For phosphatidylcholine, the area is approximately 0.71 nm^2^. For a unilamellar liposome, it can be simplified as
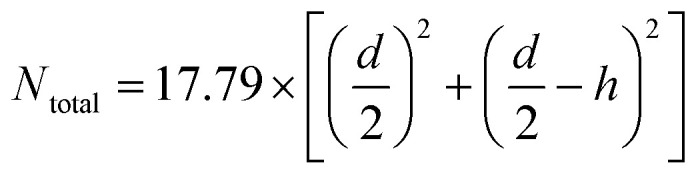


For a liposome with a diameter of 100 nm, the estimated number is 8 × 10^4^. For the U4T and lipid ratio of 122.5, each liposome has 326.5 U4T molecules on the outer leaflet. The eCpG on the liposome is 326.5, and its density is 0.01 nm^−2^.

For CpG coverage, each AuNP has roughly 100 CpG strands; thus, the surface density is 0.19 nm^−2^.

The aggregation number of the micelle was estimated to be 23, and its surface density is 0.07 nm^−2^.^44^

### *In vivo* treatment

When 6-week-old mice were injected intravenously with 100 µL of a nanoparticle solution, they were sacrificed 18 hours after injection.

### Analysis of spleen DCs

Spleens were cut into small fragments and digested with 2% FBS containing collagenase IV for 20 min at room temperature. Digested cells were centrifuged, and the pellet was resuspended in 5 mL Histopaque®-1077. An additional 5 mL of Histopaque®-1077 was upper-layered below, and FBS was layered above the cell suspension, which was then centrifuged at 1700 g for 10 min. The light density fraction (<1.077 g cm^−3^) was collected and incubated for 20 min with the following FITC-conjugated monoclonal antibodies (mAbs) for lineage staining: anti-CD3 (17A2), anti-Thy1.1 (OX-7), anti-B220 (RA3-6B2), anti-Gr-1 (RB68C5), anti-CD49b (DX5), and anti-TER-119 (TER-119). The lineage–CD11c^+^ cells in live leukocytes were defined as spleen DCs. Analysis was carried out on a Novocyte (ACEA Bioscience, San Diego, CA, USA).

### Flow cytometry analysis

Cells were washed with PBS containing 0.5% bovine serum albumin, pre-incubated for 15 min with unlabelled isotype control antibodies (Abs), and then labelled with fluorescently labelled Abs by incubation on ice for 30 min, followed by washing with PBS. Cells were analysed on a Novocyte (ACEA Bioscience, San Diego, CA, USA) and with NovoExpress software (ACEA Bioscience, San Diego, CA, USA). Cellular debris was excluded from the analysis by forward- and side-scatter gating. Dead cells were further excluded by 7-AAD staining and gating on the 7-AAD-negative population. As a control for nonspecific staining, isotype-matched, irrelevant monoclonal antibodies (mAbs) were used.

### ELISA

TNF-α and IL-6 concentrations in sera were measured in triplicate using a standard enzyme-linked immunosorbent assay kit (ELISA, BioLegend, Inc.).

### Statistical analysis

All the data from nanoparticle characterization are expressed as the mean ± standard deviation (SD). All the data from animal experiments are expressed as the mean ± standard error of the mean (SEM). The statistical significance of differences between experimental groups was calculated using analysis of variance with a Bonferroni post-test or an unpaired Student's *t*-test. All *p*-values <0.05 were considered significant. All data from animal experiments are presented as the mean ± SEM. *p < 0.05, **p < 0.01 *versus* the PBS group.

## Ethical statement

All animal experiments were conducted in accordance with the Institutional Animal Care and Use Committee (IACUC) guidelines of the Asan Medical Center. All animal procedures, including handling, housing, anesthesia, and euthanasia for this study, were approved by the IACUC of ASAN Medical Center (approval no. 2023-04-041).

## Author contributions

H. L., H. B. P., and H. K. contributed equally to this work. H. L. and H. B. P. performed most experiments. M. K., J. O. J. and A. H. conceived the project. H. K. and S. H. L. analysed data and provided feedback. All authors contributed to writing, reviewing, and editing.

## Conflicts of interest

There are no conflicts to declare.

## Supplementary Material

NH-011-D5NH00726G-s001

## Data Availability

The data supporting this article have been included as part of the supplementary information (SI). The supplementary information contains supplementary figures and tables related to the characterization of nanoparticles. See DOI: https://doi.org/10.1039/d5nh00726g.
