# Supplementary material for: CpG-induced immune responses via DNA micelles, gold nanoparticles, and liposomes
Source: Nanoscale Horiz. 2025 Nov 27;11(3):778–85. doi: 10.1039/d5nh00726g (PMC12690286; doi:10.1039/d5nh00726g)
Supplement: NH-011-D5NH00726G-s001 [file NH-011-D5NH00726G-s001.pdf]

Supplementary Information (SI) for Nanoscale Horizons.
This journal is © The Royal Society of Chemistry 2025

Supporting Information

CpG-induced immune responses via DNA micelle, gold nanoparticle, and liposome

Hongyan Li,[a, b] Hae-Bin Park,[c] Haejoo Kim,[d, e] Sang Hak Lee,[e] Andreas Herrmann,[a, b, *] Jun-O Jin,[c, *] and Minseok Kwak[d, *]

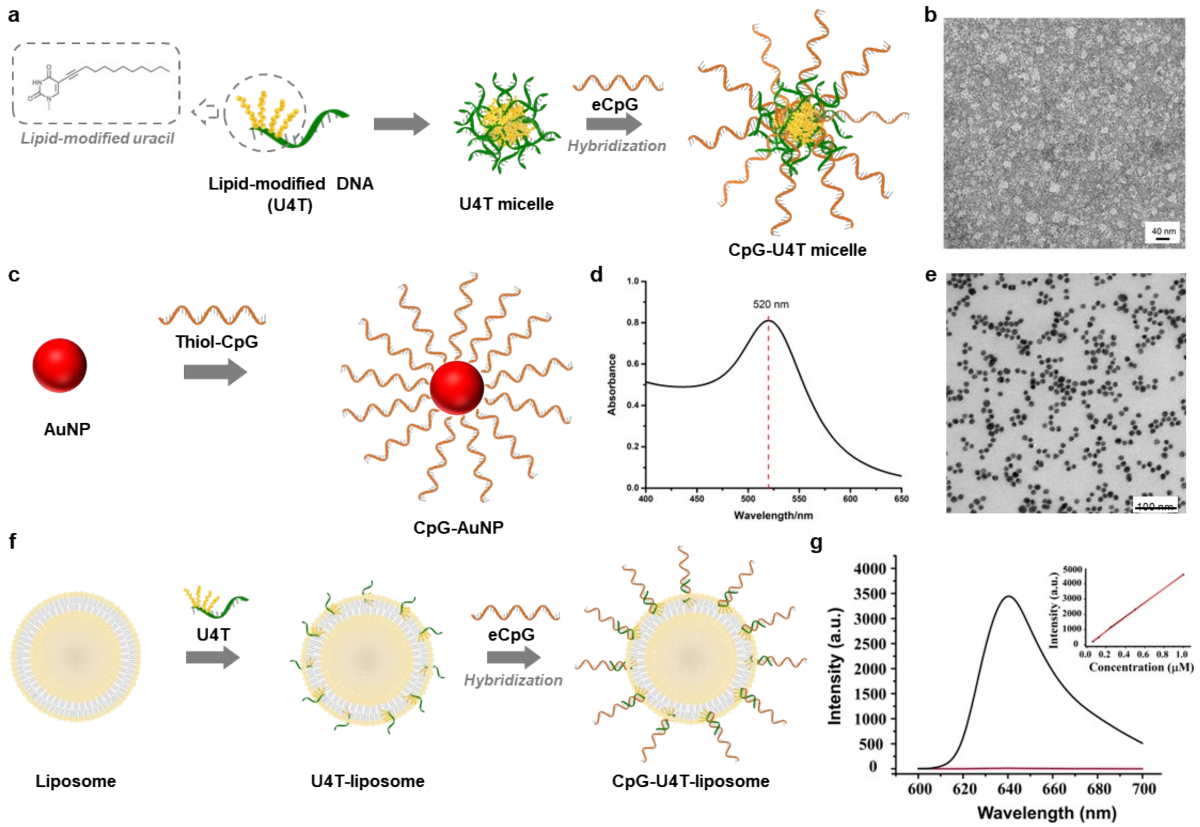

Figure S1. Immune adjuvant carriers. (a) Illustration of CpG-U4T micelle. (b) Morphological characteristics of DNA micelle by transmission electron microscopy (TEM). The TEM image showed that DNA micelle forms uniform spherical aggregates. (c) Illustration of CpG-AuNPs. (d) Absorption spectrum of AuNP. The absorption spectrum exhibited a characteristic surface plasmon resonance (SPR) peak at 520 nm, indicating the AuNPs with a size of 15 nm.[1] (e) TEM image of AuNP. The TEM image showed that the synthesized AuNPs were spherical with a uniform size distribution. (f) Illustration of CpG-U4T-liposome. (g) Fluorescence spectra of ATTO590-CpG-U4T-liposome. The fluorescence intensity of ATTO590-CpG-U4T-liposome (black) and U4T-liposome (red) was measured to quantify the amount of CpG loaded in the liposomes. The inset graph showed the standard calibration curve of ATTO590-eCpG at different concentrations, which was used to determine the amount of CpG in the liposome.

Table S1. Zeta potential values of all nanoparticles.

Nanoparticle	Zeta potential	Nanoparticle	Zeta potential	Nanoparticle	Zeta potential
**U4T micelle**	-20.3	**AuNP**	-44.8	**Liposome**	5.8
**CpG-U4T**	-31.0	**CpG-AuNP**	-26.7	**U4T-liposome**	-8.5
				**CpG-U4T-liposome**	-20.2

Table S2. Hydrodynamic diameter of nanoparticles.

Nanoparticle	Hydrodynamic diameter (nm)	Standard deviation
**U4T micelle**	8.85	1.77
**AuNP**	12.80	2.64
**Liposome**	98.85	22.15

References

1. S. Link and M. A. El-Sayed, *J. Phys. Chem. B*, 1999, **103**, 4212-4217.
